# “Mejorando Nuestras Oportunidades”: Engaging Urban Youth in Environmental Health Assessment and Advocacy to Improve Health and Outdoor Play Spaces

**DOI:** 10.3390/ijerph16040571

**Published:** 2019-02-16

**Authors:** Flavia C. Peréa, Nina R. Sayles, Amanda J. Reich, Alyssa Koomas, Heather McMann, Linda S. Sprague Martinez

**Affiliations:** 1 Mindich Program in Engaged Scholarship, Harvard College, Cambridge, MA 02138, USA; nrsayles@fas.harvard.edu; 2 Center for Surgery and Public Health, Brigham and Women’s Hospital, Boston, MA 02120, USA; ajreich@bwh.harvard.edu; 3 Alliance for a Healthier Generation, Portland, OR 97201, USA; alyssa.koomas@healthiergeneration.org; 4 Groundwork Lawrence, Lawrence, MA 01840, USA; hmcmann@groundworklawrence.org; 5 School of Social Work, Boston University, Boston, MA 02215, USA; lsmarti@bu.edu

**Keywords:** Community-Based Participatory Research (CBPR), youth engagement, environmental assessment, parks and play spaces

## Abstract

Youth can be valuable partners in community health improvement efforts. Latino youth from Lawrence, MA were engaged in research and health promotion over an 11-month period. Utilizing their knowledge of the community, youth assessed local parks and carried out evidence-based health promotion efforts to communicate community resources to encourage physical activity, nurture community ownership of parks, and advocate for park improvements. Health promotion efforts can engage youth in strategies to address critical public health issues by leveraging their unique perspective and distinct location within communities. The communications developed by the youth were distributed within the community, benefiting residents directly. Youth were motivated to engage in the project by a sense of civic obligation, and upon completing the project, they expressed that they had gained research and communication skills and were inspired to continue to support their community. Youth engagement in applied research and health promotion at the local level can provide a foundation for community health improvement efforts that are relevant for distinct communities, while fostering the positive development of youth, and nurturing community-driven efforts to help create a healthier environment.

## 1. Introduction

Unhealthy environmental conditions have contributed to the dramatic increase in chronic diseases, including obesity [1]. Communities of color and low-income communities are more likely to experience environmental exposures than their white counterparts [2]. This is particularly true with respect to the built environment, where communities of color and low-income communities overall, have less access to green space, parks, and playgrounds than white or affluent communities [3]. Characteristics of outdoor spaces and the built environment in some instances enhance opportunities for youth to engage in physical activity and in others serves as a deterrent to physical activity. For example, walk-ability and the presence of high-quality outdoor parks and play spaces can serve to catalyze engagement in vigorous physical activity, which has positive implications for overall health [4,5,6,7]. On the other hand, youth are less likely to be physically active when there are few play spaces near their homes or when parks are deemed unsafe and/or lack amenities such as lights, toilets, and drinking water [8].

Thus, the features, quality and conditions of parks in communities of color and new immigrant communities are important for understanding physical activity (PA) resources as well as possible barriers to PA, and for developing appropriate efforts to promote more optimal health. In direct response to these issues, we partnered with municipal and non-profit stakeholders in the city of Lawrence Massachusetts, and engaged local youth to conduct a comprehensive park assessment and develop a health promotion strategy. At the time of this project, Lawrence was rich in planned green space including parks and play spaces, however, youth expressed concerns related to sanitation and safety in said spaces [9].

This paper highlights the value of including youth in public health research and decision-making that affect their lives and their health, but which until only recently has historically left out their voices [10,11]. Youth can be valuable partners in community health improvement efforts and can be engaged to advocate for park improvements and communicate community resources to encourage PA. During the grant planning phase our adult community partners arranged a set of conversations with groups of youth, who helped us to conceptualize a research and action study focused on local parks and play spaces. The goal of the project was to utilize the research as a basis to engage a group of local youth as leaders in community health promotion, reflective of priorities defined by the group. Similarly, the project approach sought to leverage the youth’s location and expertise as community members to personalize health promotion efforts, and craft messaging they felt was important for their community to hear.

A brief background on participatory research and the benefits of youth participation is provided. We then present a model for engaging youth in collaboration with municipal leaders and non-profit stakeholders, in research and action focused on increasing access to parks and play spaces in Lawrence, MA, a majority Latino municipality. We will outline the assessment methodology, results, and present in detail the health promotion strategy developed and implemented by the youth over the course of their involvement. Drawing directly from the health promotion and advocacy materials developed and publicly disseminated by the youth, we will then describe impact the project had on the youth. This paper adds to a growing body of literature on youth engagement in public health research and action efforts, by illustrating how youth engagement in applied research and health promotion at the local level can help bridge the gap between research, public policy, non-profit work, community and youth development to help create a healthier environment.

## 2. Background

There are multiple benefits associated with community participation in research [12]. These range from increasing a study’s sustainability and ensuring it is reflective of local priorities [13] to building both researchers and community capacity and facilitating resource sharing [14]. Although the engagement of youth in research has a long history in fields such as education, community psychology and social work, the inclusion of youth in the research process is a fairly recent development in public health, even in the context of community-based participatory research (CBPR) efforts, which have primarily privileged adult voices, engaging been adult stakeholders and residents, as partners in research [15]. Youth are therefore a community asset that has often been overlooked in public health research.

The body of literature on youth participation in research indicates there are benefits for youth, adults, organizations and the broader community [11]. However, research suggests that all youth do not have equal access to opportunities for community engagement, with differences by race, ethnicity, and social class [16]. Youth are most likely to become engaged in their communities when they are knowledgeable about issues and methods for action, and when they are asked by someone to join an organization or attend a meeting; similar to the format in which youth became involved in this project [16]. Importantly, after-school programs can be important contexts for adolescent development, particularly among youth of color and low-income youth [17]. As research into youth’s experiences with these activities increases, evidence suggests that the type of engagement, the intensity, duration, and multiple socioeconomic differences effect development outcomes [18]. Youth identified three valuable features of such programs: supportive relationships with adults and peers, safety, and opportunities to learn [17]. These contributions are particularly important for minoritized youth in under-resourced communities, as previous studies have found that youth participatory research that engages minority youth in poor communities in research regarding health in their own communities can be particularly successful, offsetting substandard education with opportunities for positive youth development [19].

Youth participation in research and action efforts to improve local living conditions and health can increase: civic mindedness, community engagement, perceptions of power to make change in the community and resilience [20,21,22,23]. Participatory research models that engage youth emphasizes a sense of ownership and control for the youth, promotes social and political engagement, and focuses on ameliorating problems identified through the research [24]. One assessment of youth-led research among an ethnically diverse group of urban middle school students found that youth outcomes included skill development in research, communication, and advocacy, positive ethnic identity and sense of purpose, psychological and political empowerment, and expanded social networks [25]. A Photovoice project that engaged an ethnically diverse group of youth in a low-income California community [26], found that action projects that engage youth with their social environments can foster critical thinking skills development among young people. Lastly, youth who participate in health research and action demonstrate a more holistic understanding of health and elements of the built environment that influence wellbeing; the research process; and critical thinking [21,23,27,28,29]. Moreover, by engaging young people as partners adults learn to value their perspective and develop a fuller awareness of youth as a community asset [30,31,32].

## 3. Mejorando Nuestras Oportunidades (Improving Our Opportunities): A case study

This project was unique in that it was invited by the Mayor’s Health Task Force (MHTF) for the City of Lawrence, with support from the Mayor, Community Development, Police, Public Works, and Recreation Departments. Therefore, community stakeholders brought academic researchers to the table. The MHTF serves as an advisory board to the mayor and places public health issues high on the city’s political agenda. The project implemented was a collaboration between Tufts University, the Lawrence Community Development Department (CDD), and Groundwork Lawrence (GWL). The CDD is the city’s chief agency for community planning and urban development, and focuses on improving the quality of life in Lawrence. GWL is a local non-profit urban environmental and youth development organization and part of Groundwork USA. GWL addresses environmental issues through on-the-ground projects focused on youth, empowerment, sustainability, economic and social well-being, particularly by nurturing the potential of the city’s youth who live in neighborhoods that have experienced decades of decline in their physical and social environments.

With a focus on linking inquiry and action, this project entailed assessing parks and playgrounds to develop local health promotion and advocacy efforts to increase PA. Through a CBPR partnership with municipal and non-profit leaders, local youth were employed as researchers by the university. Although youth are not typically involved in public health promotion and advocacy efforts, this project sought to involve youth throughout the project as their perspective and intimate knowledge of Lawrence would help guide the research and ensure a high degree of research integrity. Because Lawrence is a city fiercely committed to youth leadership, having a research team comprised of Lawrence youth, and having the youth out in the community collecting data (as opposed to employing undergraduate student research assistants), was a natural fit for this project that would help build trust and ensure community buy-in. Having local youth as part of the research team, would also provide the youth with a valuable experiential learning opportunity, underscoring the emphasis on reciprocity the project was rooted in.

We have previously reported findings from the youths’ assessment of Lawrence’s parks, including pictures of the parks taken by the youth, and the incivilities present in the parks identified by the youth [9]. Activity levels in the parks have also been presented previously [33], as have findings of how physical activity levels intersect with the presence of incivilities in Lawrence’s parks [34]. In this paper, we describe how to engage youth in action-oriented community assessment, as well as the impact on the youth when they engage in participatory action research projects. To provide some context, we will first provide some background about Lawrence. Second, we will describe the methodological and analytic methods. Third, we will describe the youths’ health promotion strategy. Lastly, we will present findings from the interviews and our examination of the youth’s health advocacy materials.

### 3.1. About Lawrence

When this study was conducted, Lawrence was ranked as the poorest city in Massachusetts. In June 2013 it had the highest unemployment rate (14.1%), almost double the state average that year (7.1%). In the last five years, the unemployment rate has improved dramatically, to 5.2% in October 2018, however Lawrence continues to lead the state in unemployment, ranking second highest in unemployment behind Springfield, MA [35]. Like many former New England manufacturing towns, it has suffered economically due to the economic shifts and labor market changes that began in the latter half of the 20th century. An immigrant city of 72,000 residents, in 2013, Lawrence was nearly 73% Latino, predominantly Dominican and Puerto Rican, and 36% foreign born. Over 74% of residents spoke a language other than English at home. The per capita income in Lawrence was $17,068 in 2013, and over the next five years, that number just barely rose to $18,069, while the estimated national average in 2017 was $31,177. While our research was conducted, over 90% of students qualified for free or reduced-price lunch [36].

**Figure 1:**
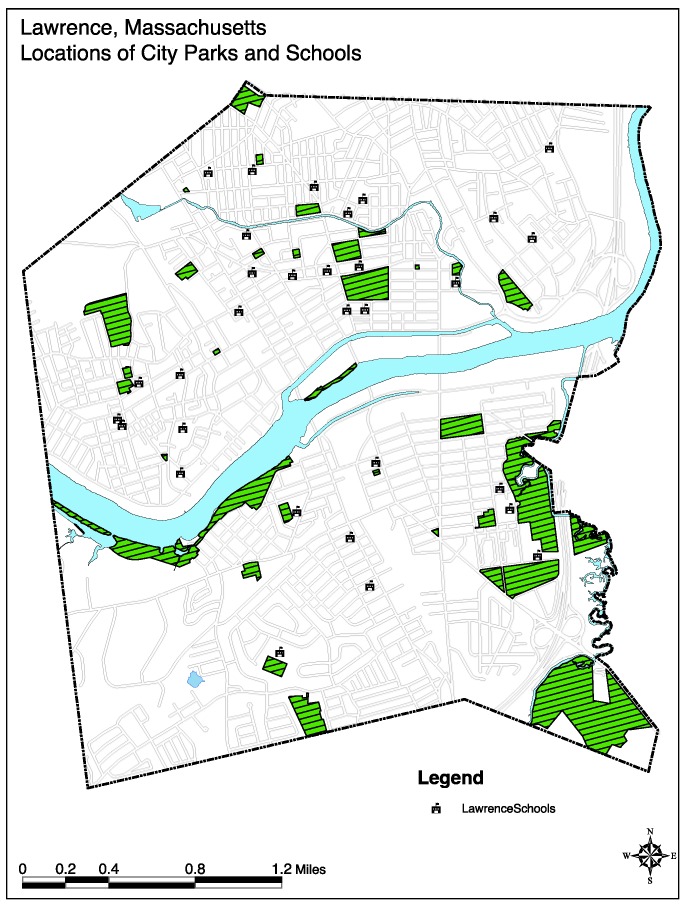
Locations of City Parks and Schools, 2004 [38].

There are a number of parks in Lawrence, and the city has many playgrounds, pocket parks, and open green spaces, as well as athletic fields (see Figure 1). In this context, the city of Lawrence prioritized improving outdoor and recreational spaces, and had implemented an open space plan, which included multiple park renovations, and the construction of new baseball fields, open spaces, and green spaces [37].

The MHTF actively seeks to engage youth by involving them in community health efforts, and the city is exploring how policy-driven community engagement can improve the public health, particularly among young people. Early conversations with municipal leaders and youth highlighted two priorities, a focus on access to recreation and outdoor play spaces and youth workforce opportunities. As such, we focused on employing local youth as the research team, a strategy that was recommended by youth we met with during our initial grant planning phase. This allowed us to ensure youth leadership and decision-making throughout the project.

### 3.2. Methodology

The environmental health assessment at the basis of this project that provided the foundation for the youth’s health advocacy efforts entailed observational assessment to understand the quality and condition of parks and playgrounds [9].

#### 3.2.1. Youth Researcher Recruitment

In an effort to identify local youth researchers, paper applications as well as an electronic application form were distributed by our community partners. Applications were disseminated at Mayor’s Youth Task Force Meetings, public schools, a community college, and an employment organization. The application explored youth interests in community engagement, motivations for applying to the position, and previous work experience as well as work eligibility. Demographic data was also collected, and youth were as to provide the names of 2–3 references. Over the course of three weeks a total of 28 completed applications were received. Two researchers reviewed the applications and using a rubric selected the top 14 candidates who were invited to participate in and in-person interview held at the public library. Interviews further explored participant motivation as well as ability to attend weekly meetings, and to commit for the duration of the project. Finally, candidates (*n* = 6) were invited by the researchers to join the project. Of the six youth selected, two identified as male and four identified as female; the age range was 16–20. All identified as Latino (three Dominican, two Puerto Rican and one Mexican). Youth represented a number of educational settings that included alternative and international high schools, as well as the local community college. One youth was disconnected from school. All but one of the youths remained engaged with the project for 11 months (*n* = 5). The youth who left the program reported needing a full-time job to support a newborn.

#### 3.2.2. Youth Researcher Role

Youth from Lawrence were recruited as research assistants in April 2010 and were involved through February 2011. The youth researchers were responsible for informing the design and implementation of the environmental assessment, as well as fieldwork protocols, analysis of assessment data, local dissemination, outreach and advocacy. At the project launched with a one month training period where the youth participated in a series of in-person trainings focused on health equity and the social determinants of health; social science research, field research and human subject’s ethics. In addition, youth learned about assessment and were trained specifically in observational parks assessment methods using the System for Observing Physical Activity and Recreation in Communities (SOPARC) [39] and the Physical Activity Resource Assessment (PARA) [40] tools. Over the course of the project period youth were introduced to additional topics during weekly meetings which included, dissemination and health promotion. Trainings were provided for youth researchers and adult partners by the investigators. Meanwhile, sessions related to municipal dynamics, park investments and community assets were provided by adult community partners and as the project progressed youth advised on dissemination as well as local priorities and engagement strategies. Teamwork, co-learning, leadership, community engagement, decision-making, writing, public speaking, and the benefits of the project for the community were emphasized throughout. The youth participated in regular weekly research team meetings and independent work that entailed 6 hours of work per week over an 11-month period. Park assessments were conducted for six months from May to October 2010. The youth developed and lead health advocacy efforts in the city for seven months starting in Aug 2011. Each youth received a $200 monthly stipend. Further details on the youth recruitment and work of the youth is reported elsewhere [9].

#### 3.2.3. Environmental Assessment

The youth assessed in 23 parks under city jurisdiction. Parks under the jurisdiction of the state where excluded by the adult community partners. After the initial training, adult community partners and researchers presented youth with a list of all municipal parks as well as maps. Youth divided the lists and using the maps identified the parks they were familiar with. Youth excluded pocket parks, or small patches of grass at intersections and corners. In addition, youth excluded a park that none of them were comfortable visiting due to safety concerns. Once the selection of parks was finalized youth in groups developed data collection schedules and divided the parks. The youth systematically assessed the quality of and conditions in parks using the PARA tool [40], and the SOPARC [39] was used to assess utilization. Both the PARA and SOPARC were identified by the adult partners and researchers at the time of the initial grant submission. The youth used paper assessment forms and filled out a single assessment form by hand for each observation. These data were then entered into a database for analysis. The PARA focuses on elements of the physical environment including, incivilities (e.g., presence of litter, graffiti and vandalism, evidence of alcohol use), and amenities (e.g., bathrooms, drinking fountains), and features (e.g., equipment, courts), ranked on a scale of 0–3 (none, poor, mediocre, good). Data on park utilization include the estimated age and gender of each individual in a park at each observation point, the type of activity each person was engaged in, and perceived PA level on a scale of 1–3 (low, medium, high). In addition, the youth used photography to document park conditions as part of their assessment. The youth had discretion about when to take pictures and what to document visually. Each youth was provided with a digital camera for their use throughout the project. Observations were conducted three times a day during the morning, afternoon and evening, on one weekday and one weekend day each week, on consistent days throughout the data collection period. In cases where public health hazards were identified, such as hypodermic needles, youth followed a protocol that involved contacting adult partners at groundwork who reported incidents to the authorities.

#### 3.2.4. Evaluating Impact on Youth Researchers

To assess the impact of the project on the youth researchers, we: (1) conducted interviews with the youth researchers, and (2) examined the health promotion and advocacy materials the youth developed and disseminated based on the environmental assessment results. The purpose of the interviews was to understand how being a youth researcher impacted the youth. Specifically, we examined how the project impacted the youth’s confidence, perceptions of ability to make change, sense of community and community engagement. Interview questions also explored the types of skills and resources the youth gained through their involvement with the project. Ongoing self-reflection by the youth regarding their involvement with the project, their thoughts on the project’s impact on the community, as well as their personal growth and development, were integrated into their communications with the community. To more deeply understand the project’s impact on the youth, the materials written by the youth were therefore analyzed to extract text related to youth development and community engagement. The study was approved by the Tufts University Institutional Review Board (at the time of the study the investigators were on the faculty at Tufts University). Both a youth assent and parental consent, when applicable, was obtained for participation in the study. Parental consent was administered in English and Spanish.

### 3.3. Qualitative Analyses

Audio recordings of interviews were transcribed and text files generated for analysis. Text file transcripts were analyzed by hand, reviewed and double-coded by two researchers. Content analysis was systematically employed by both researchers to categorize and label the data. The researchers then discussed the identified categories and worked together to identify key themes and specify codes. The interviews were coded by two researchers to ensure inter coder reliability. The researchers then met to review the coding and key findings.

Based on findings from the interviews, qualitative analysis techniques were applied to project documents to examine if the key themes that emerged in the interviews were evident in the materials developed by the youth. Two researchers each applied the codebook to the health promotion and advocacy materials the youth developed. The researchers then met to review the coding and discuss findings. The researchers then matched findings from the interviews with findings from the examination of the youth’s materials.

Although the youth were involved in the analysis of the park assessment data, the youth were not involved in the analysis of qualitative data related to their engagement with the project presented here. Because the youth’s involvement with the project, and compensation, had long come to a close, it was not possible to involve the youth in thus component of the project.

### 3.4. Health Promotion Strategy

As described in detail elsewhere [9,33,41], the youth observed health and safety issues across Lawrence’s parks. Forty percent of individual observations in Lawrence’s parks indicated, to varying degrees, the presence of litter, graffiti, vandalism and evidence of alcohol (i.e., empty beer cans) in the parks. Litter and graffiti/tagging were the most common incivilities observed. Additionally, sex paraphernalia and auditory annoyances were observed, but much less frequently. Only one park had unlocked bathrooms with flush toilets and sinks, and only two parks had functioning water fountains. Based on the results, the youth identified sanitation and safety (graffiti was interpreted by the youth as an indicator of gang activity) as the issues to address through advocacy and health promotion efforts [9].

Lawrence is a vibrant Latino majority city that has tremendous assets; notably, it is a highly collaborative and engaged city where residents and stakeholders across sectors work hard to make Lawrence a great city to live, work, learn, and play [9]. However, at the time of this Lawrence also had a high crime and severely under-resourced sanitation department. As the youth reported, “Lawrence is often viewed in a negative light”. Because of this, the youth, GWL and the CDD felt that dissemination efforts should focus on promoting positive aspects of the parks and ways residents might engage in them. Youth additionally noted that parks were overlooked and often unknown by the city’s residents. Given their ideas and expertise, youth were given space to shape dissemination efforts with the support of community partners who felt youth voices would draw more attention from the community than those of adults. Community health improvement initiatives are often designed by officials and experts who are frequently “outsiders” not from the target community; youth are rarely engaged. In such instances, an understanding of community context is sometimes lacking. Youth in this instance proved to be an important bridge between our partners and the broader community, which allowed us to avoid the types of unidirectional messages that are typical in public health communication [42].

With this foundation, the youth chose a variety of personalized communication materials that reflected their experience as Lawrence youth would be the best way to reach the public. Through a community newsletter, newspaper articles, and a report on the city’s parks they created and authored, as well as through public meetings and presentations, the youth shared what they learned through the project in their own words as a springboard to provide the community with information on city resources, and give recommendations for the youth, residents, and the city government regarding parks, PA, health and obesity in the city. Youth sought to get people moving and promote a sense of ownership and shared community responsibility for the city’s parks and playgrounds, which they felt was desperately needed. For the youth, a lack of ownership and shared responsibility of open spaces is what contributed, in large part, to the sanitation and safety issues that were a problem in so many parks [9]. They also sought to reach policy makers and city leaders who were in positions to act on the research findings and their recommendations.

#### 3.4.1. Newsletter

**Figure 2:**
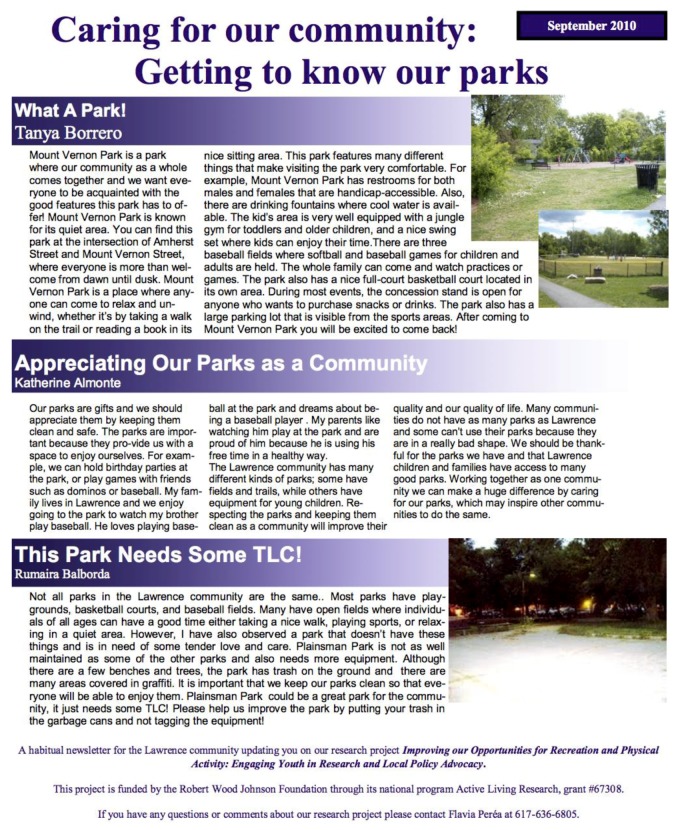
Newsletter: Caring for our Community.

The youth wrote a monthly newsletter, *Caring for our Community: Getting to Know our Parks*, which was published bilingually in English and Spanish. The newsletter emphasized the importance of PA and the benefits to health, particularly for children and youth, by promoting the community and parks. The youth designed the newsletter to emphasize stewardship and ownership of public parks while promoting the community and health benefits of parks. For example, as stated in one of the youth’s newsletter articles presented in Figure 2:

Our parks are gifts and we should appreciate them… parks are important because they provide us with a space to enjoy ourselves… The Lawrence community has many different kinds of parks. Some have fields and trails, while others have equipment for young children. Respecting the parks and keeping them clean as a community will improve their quality and our quality of life. Many communities do not have as many parks as Lawrence and some can’t use their parks because they are in a really bad shape… Working together as one community we can make a huge difference by caring for our parks, which may inspire other communities to do the same.

[43]

The newsletter also highlighted the city’s many wonderful parks, as well as those with safety or maintenance issues with suggestions for how to address them, but always through an asset-based lens. For example:

There are some parks in Lawrence that are not treated with care, and we intend to improve this situation. Many parks have beautiful open areas that encourage kids to use them, whether it’s walking on a trail or playing on a jungle gym. Sullivan Park is one park that needs a little TLC. We have observed a lot of trash scattered around the area and a lot of graffiti. The park is very well equipped with a tennis court, two full basketball courts, benches, chairs, swings, and a jungle gym; but, the area and the equipment are being treated very poorly and that is unfair to both the people who currently use the park and people who would like to use it in the future. It is really important to use the parks in a positive way for now and for the future!

[44]

As this excerpt illustrates, the youth’s messaging sought to increase awareness in the community about parks that were not well cared for, while encouraging people and families to take advantage of the parks. The articles were accompanied by photographs of the parks taken by the youth.

Print copies in both languages were made available to the public throughout the city including at the Public Library, City Hall, and a local employment agency. They were also disseminated locally electronically through the MHTF listserv as well as through stakeholder networks. To increase visibility of the project in the wider Boston area, the newsletters were also electronically disseminated through resources at Tufts University. By using these mechanisms, it was possible to reach a wider audience comprised of both researchers, lay people, and community organizations engaged with academic institutions in the Boston area.

#### 3.4.2. Newspaper articles

**Figure 3:**
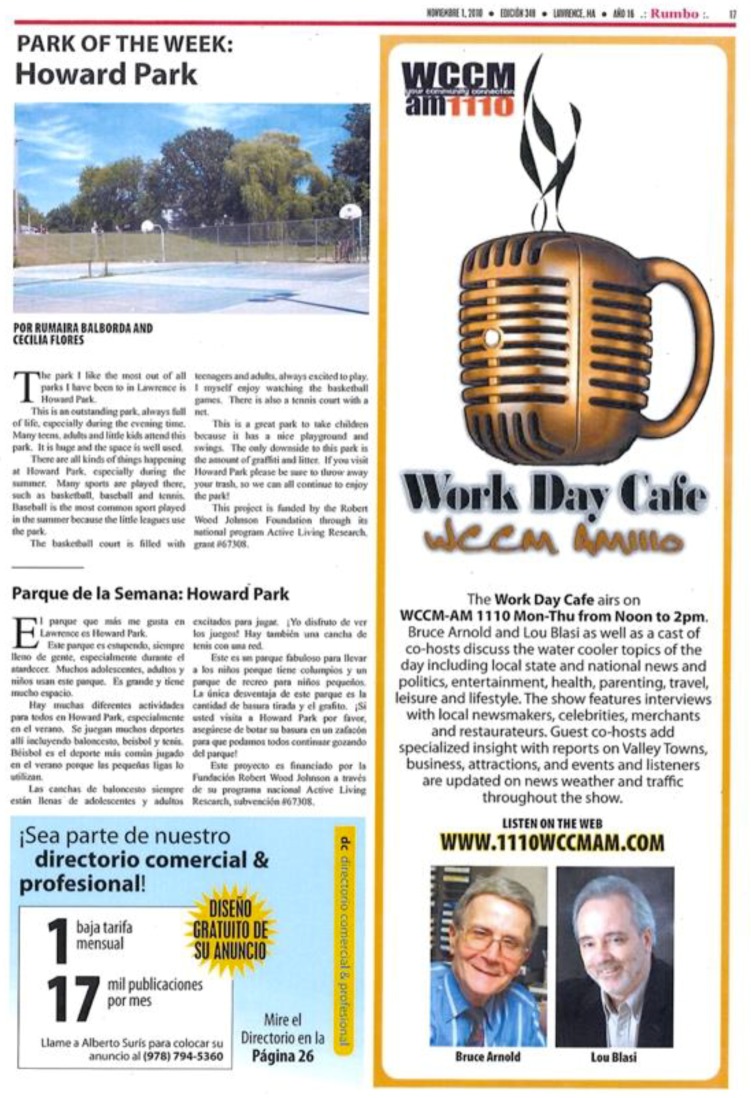
“Park of the Week” from November 2010 edition, Rumbo News [45].

The youth health promotion strategy also included working with a local English/Spanish bilingual newspaper, *Rumbo*. *Rumbo* emphasizes positive articles about Lawrence, is available in print throughout Lawrence and the surrounding region, as well as free online [33]. Lawrence is a community where many people continue to get their news from printed newspapers, particularly smaller local and regional papers that are widely read. The youth wrote a short bi-monthly article for *Rumbo* entitled “Park of the Week” to highlight one of the city’s great parks, which included a picture of the park. For example, as described in one the youth’s articles for *Rumbo* presented in Figure 3:

[Howard Park] is an outstanding park, always full of life… Many teens, adults, and little kids attend this park. It is huge and the space is well used… The basketball court is filled with teenagers and adults, always excited to play. I myself enjoy watching the basketball games… This is a great park to take children because it has a nice playground and swings.

[45]

Although there are safety issues evident in Lawrence’s parks [9], through these short articles the youth sought to combat negative and distorted perceptions of the parks. By drawing attention to the many features of the parks, the youth aimed to publicize them as fun places to spend time outside. Given the youthfulness of the city, and the large number of families, the youth wrote these articles with families and young people in mind.

#### 3.4.3. Report and Recommendations

At the culmination of their involvement with the project the youth put together a final Report on Lawrence’s Parks [46]. Developed for local dissemination, the purpose of the report was to share the results of the park assessment with the Lawrence community with a focus on targeting local leadership and policy makers to support and continue to improve parks and playgrounds. The report emphasized the positive aspects of every park assessed, and each park was scored on five criteria: recreation factor (example: fun, relaxing things you can do there), cleanliness, safety, amenities, condition of existing features, and an overall score. The grading system was developed by the youth research team for the report, which contained specific recommendations developed by the youth for youth, residents, and the city government for how to improve the condition of the parks and increase their utilization. The youth’s recommendations were centered on increasing community ownership of parks, and focused on ways to improve the parks by fostering a sense of community through parks promotion, for example:

- *Youth should* get more involved; and be respectful, caring, considerate and loving of the parks.
- *City government should* publicize the parks- tell residents about all of the exciting things they can do in the parks; improve the safety of the parks.
- *Residents should* take ownership of the parks and treat them as you would your home; create murals instead of drawing graffiti with foul words [46].

**Figure 4:**
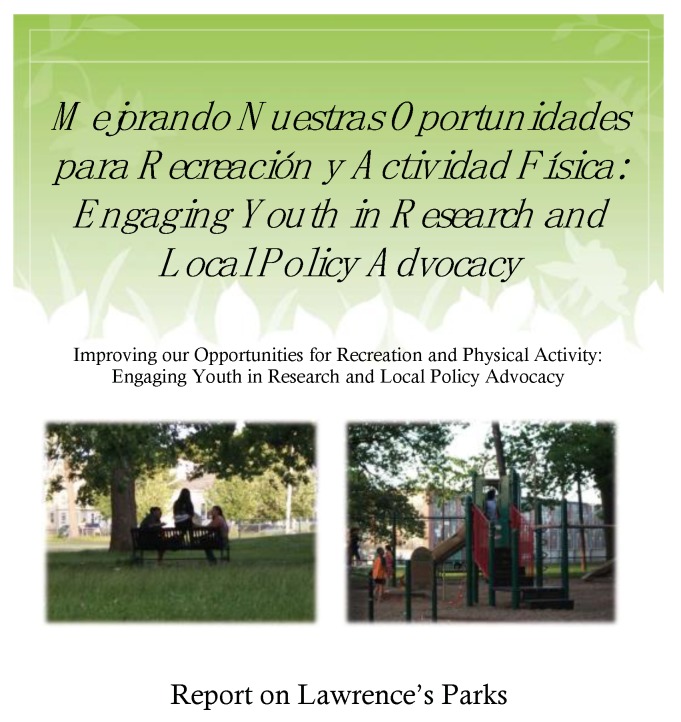
Cover page of final Report on Lawrence’s Parks [46].

The report, the cover of which is presented in Figure 4, was disseminated throughout the city, in particular to key stakeholders such as the MHTF, mayor, city council members, local department heads, and non-profits involved in health and environmental issues. The report was also made available online and disseminated electronically through the same channels as the newsletter.

### 3.5. Interview Findings

The youth participated in an interview, and responded to a series of questions regarding their specific experiences as youth researchers and, more broadly, engagement in the Lawrence community (the questionnaire is given in Appendix A). Overall, the youth shared consistently positive comments regarding their work and involvment, skills gained, and their impact on Lawrence parks and the community. Negative feedback was limited to program logistics (i.e., transportation and meeting times), and the concern that local parks might not be appreciated or appropriately cared for. As detailed in Table 1, key themes were organized into two categories: communication and awareness. Communication was an important component of the program and developed via multiple formats: oral and written communication, learning to work on teams and use data to share findings.

Much of the feedback youth provided regarding the experience was related to communication skills:

“…being able to go out and do presentations, experience with public speaking, meeting great people, connections and opportunities.”

In response to the question, “What has been the most valuable part of being a research assistant?” youth also cited data use:

“The fact that we put the data together….learning how to collect data and having experience with that work.”

In regards to the theme of awareness, youth described the experience as promoting awareness personally (learning more about the parks in their own community) in addition to raising awareness among peers, community members, and Lawrence leadership. Youth expressed optimism that their work could motivate others to use and care for parks. One youth described the project’s impact on Lawrence parks and the community,

“…Probably give people a different picture of the parks, many people have the opinion that Lawrence is not a good or safe area, the parks could change their minds and also draw attention to the places where more work and help is needed.”

Youth who were engaged in the project gained a better personal awareness of local park resources. In response to the question, “Do you feel more connected to the community now than you did before the project?” the overall consensus was affirmative. Youth cited,

*“…Seeing the beautiful parks that (we) didn’t know about,”* and

“…now (we) go and hang out there in our free time.”

Finally, interview responses indicated the youth felt their work had an impact in Lawrence, and noted that when other people saw what they were doing, community members would be motivated to help and create even more change. Youth described the community as more aware of the parks and pointed out that improvements had already begun.

### 3.6. Impact on Youth

Through the health promotion materials and ongoing reporting to the community the youth often reflected on their experiences as researchers. The ongoing self-evaluation integrated into the youth’s writing presented a rich and nuanced source of information to examination of the impact of the project on the youth. Through qualitative analysis of the youth’s communications and advocacy materials we found they described a number of benefits associated with their participation in the project in the areas of communication and awareness, which bolstered findings from the interviews. Youth described a sense of purpose and agency; they shared that their initial motivations for getting involved with the project was related to a sense of civic obligation, which grew over the course of their involvement.

The reason why I was part of this project was because I wanted to give back to my community. I also wanted to show my city that there are people who care about Lawrence… This project motivated me to do more in my city.

Giving back to my community by doing projects such as this one can make this community better… this project is valuable for the community because people have the opportunity to become more united by doing something for their community.

They also shared how the work they carried-out impacted them personally. They cited the skills and self-confidence they developed, as well as their ability to work collaboratively in a team through the project

Being part of this program gave me the opportunity to learn new skills that will be useful in my future career. I have become more confident in myself after speaking in public, learned research skills and how to work as a team.

I have learned a lot from this project especially how to work in a team… this project improved my research skills and my ability to speak in front of an audience.

Lastly, the report was a tool for the youth to advocate for the importance of parks and being physically active:

The parks in Lawrence offer opportunities to spend time outdoors and to exercise. With the high obesity rate in Lawrence, informing people of the great resource we have in our parks was something I really enjoyed… There are parks all around our community and in our neighborhoods that give us the ability to live healthier lives.

Evident in the youth’s health promotion materials is a clear sense of the youth’s awareness of Lawrence’s strengths and the importance of local outreach. Their positive outlook, sense of empowerment, confidence, and perceptions of their individual ability to make change is palpable. Based on the youth testimonials and interview findings, this community based research experience was intellectually, socially, and personally transformative.

## 4. Discussion

Community engaged research poses questions about who has ownership, decision making power, and benefits from the work. The approach we utilized in Lawrence is one example of how action-oriented research can benefit youth by providing them with work and leadership experience and making connections to resources they might not have access to. Our findings were consistent with the literature in that engaging youth in public health improvement can provide youth with important opportunities for growth and development [47]. In addition, we similarly found that youth involvement strengthens public health and community development efforts [23,48].

Because of the Lawrence community’s recognition of youth development as an integral component of any public health improvement effort in the city, the youth were uniquely positioned to engage in city-wide health promotion. The approach employed in Lawrence is a model for how youth civic participation and capacity building can be integrated into CBPR projects, and can nurture a sense of social responsibility while provide young people with a sense of ownership of the process and outcomes. The impact of the project on the youth is consistent with previous studies which have found youth-engaged initiatives lead to increased ownership and empowerment among young people, and nurture leadership skills [21,49]. As with other studies where youth used photography for their research [50], we found the youth, as they themselves articulated, experienced an increased sense of agency through their work on the project, which was evidenced by greater self-confidence, improved communication abilities, as well as greater awareness of their environment and the implications of neighborhood and community conditions on residents

Engaging youth in research can provide a variety of positive developmental opportunities for participants, and the project provided the Lawrence youth with an opportunity to develop valuable skills. Consistent with previous studies [10], the youth learned about the research process, data collection, analysis and interpretation, while gaining public speaking and presentation skills, and learned how to advocate for issues that are important to them. Moreover, the youths’ confidence, motivation for learning, and desire to be more involved in their community were poignantly evident. The youth’s excitement for a new opportunity, gaining new information, and the pride and validation they felt from being heard and feeling useful was clear, and echoes previous findings about the number of benefits to youth when they actively participate in research activities [51,52]. Previous studies designed to involve young people in health promotion have identified the opportunity to be useful by helping others and pride in one’s community as the two main benefits for youth research collaborators [52], and the Lawrence youth’s sense of pride, in themselves and their community was palpable, as was how the work strengthened their connections to their community. This bolsters previous research which has established that these kinds of outcomes among youth engaged in research can lead to their desire to change negative circumstances, and the development of social and political networks that could serve as resources for action [50].

Getting youth involved in community health improvement efforts benefited the wider community as well. The MHTF was excited by the study’s potential to inform local policy and decision-making, as well as the value placed on cultivating leadership skills among youth. The Recreation, Public Works and Police Departments appreciated the focus on engaging youth because it would help foster greater appreciation for the parks and instill a sense of social responsibility, which the Chief of Police noted were the bedrock of a safe and healthy community. All partners felt the work would help to encourage utilization and care of outdoor spaces in the city, as well as more engaged youth. These themes illustrate the importance of addressing socio-environmental factors as part of efforts to address critical public health issues.

While the youths’ communications work was aimed at promoting community change relating to the use of parks and green spaces, the results of this research may have future implications for city planning, where systematic change can be achieved through a commitment to improving the environmental resources available to poor and minority urban communities. The Lawrence youth indicated concerns related to poor sanitation in their parks, among other concerns. This issue could be remediated with more commitment from state and city departments to improving the conditions in Lawrence parks. Literature indicates that a recent decrease in local and state funding for parks nationwide has exacerbated inequalities between parks in poor, minority community and those in richer, majority white areas [53]. In addition to community engagement and collective ownership of parks and public spaces, local and state planners should prioritize funding and resources for parks and green space as a result of research produced by CBPR methods.

Our study did have limitations. Firstly, the impact of the youth’s communication and advocacy strategies were not evaluated. Although it was not possible within the context of this project to assess impact on the community, a key limitation is that we do not know if the parks were impacted by the work of the youth, or how residents’ attitudes or perceptions of the parks and PA may have changed. There were also limitations to our youth engagement approach. Firstly, the youth were not involved in the analytic work presented here; the structure of the grant and the timeline of the project precluded their ability to participate. Additionally, although having youth in a leadership role and the youths’ ownership of the work were central to this project, the research was led by academic researchers and local stakeholders; youth did not have decision making powers. The power relationships between the youth and the adults on the project were therefore embedded in the project’s structure. Although the youth did the research and made recommendations, they did not have the ability to act on their research findings and bring them to fruition. Although this is a limitation of a grant funded project with specific start and end dates, it also highlights the essentially advisory role of the youth and their inability to decide municipal initiatives and direct spending at the local level. In some ways, this reflects some of the challenges of giving up and sharing power over the research as well as the action embedded in the research in the context of CBPR projects, both in the academic and community contexts. Although participatory and community-driven, the power to decide participation and the research focus rested with the adult project partners. Put simply, the parameters for the project were pre-determined by adults who invited the youth to join the project. To engage youth more deeply in research about their communities, future youth engaged CBPR projects should endeavor to engage youth in decision making roles along the entirety of the research continuum, from project conceptualization, to design, implementation and evaluation, as well as both community-based and scholarly dissemination.

## 5. Conclusions

Partnerships can provide an opportunity for people and entities that are not usually engaged in public health efforts to become part of the process. By leveraging their unique perspective and distinct location within communities, health promotion efforts can engage youth in strategies to address critical public health issues. This benefits the youth as well as the community. Engaging youth in environmental health improvement efforts can nurture the positive development of youth by cultivating leadership skills, encouraging civic participation, fostering a sense of social responsibility, and providing youth with learning opportunities to acquire important skills and knowledge. Engaging youth in efforts provides an opportunity to promote collaboration and help to surmount the tension that often characterizes relationships between the academic, government, and non-profit sectors- youth can help to build valuable relationships. Such synergistic strategies can provide the foundation for local public health efforts that are relevant for distinct communities, while building capacity at the local level and nurturing community-driven efforts to reduce disparities and promote more optimal health.

## Figures and Tables

**Table 1:** Categories and themes from interviews.

Communication	Awareness
Oral (i.e., public speaking) and written (i.e., newsletters)	Learned of community strengths and issues (including limits of community support)
Networking	Learned of local parks, initiated community conversation
Teambuilding, working in groups	Motivation to create changes in community
Data: collection, use, and reporting	

## Appendix A

Interview Questions for youth:

- Would you describe your experience being a research assistant as mostly positive, negative, or neutral? Why?
- What has been the most valuable part of being a research assistant?
- What has been the least valuable part of being a research assistant?
- Do you feel more confident in your ability to conduct research and follow through on tasks?
- Do you think this project will have an impact on the Lawrence parks and community?
- Do you feel more connected to your community now than you did before this project?
- Are there any skills you learned by being a research assistant you think you will use in the future?
- Do you feel like you have more resources available to you for the future?
- Have your career goals changed after being a research assistant?
- Do you think you have made an impact on the Lawrence community by participating in this project?
- Would you do this type of community work again in the future?
- Is there anything else you would like to share about your experience as a research assistant?

## References

[1] Brisbon N., Plumb J., Brawer R., Paxman D. (2005). The asthma and obesity epidemics: The role played by the built environment—A public health perspective. J. Allergy Clin. Immunol..

[2] Faber D.R., Krieg E.J. (2002). Unequal Exposure to Ecological Hazards: Environmental Injustices in the Commonwealth of Massachuesetts. Environ. Health Perspect..

[3] Wolch J.R., Byrne J., Newell J.P. (2014). Urban green space, public health, and environmental justice: The challenge of making cities ‘just green enough’. Landsc. Urban Plan..

[4] Babey S., Hastert T.A., Yu H., Brown R. (2008). Physical Activity among Adolescents: When Do Parks Matter?. Am. J. Prev. Med..

[5] Cohen D.A., McKenzie T.L., Sehgal A., Williamson S., Golinelli D., Lurie N. (2007). Contribution of Public Parks to Physical Activity. Am. J. Public Health.

[6] Hoehner C.M., Brennan Ramirez L.K., Elliott M.B., Handy S.L., Brownson R.C. (2005). Perceived and objective environmental measures and physical activity among urban adults. Act. Living Res..

[7] Roemmich J.N., Epstein L.H., Raja S., Yin L., Robinson J., Winiewicz D. (2006). Association of access to parks and recreational facilities with the physical activity of young children. Prev. Med..

[8] Sallis J.F., Bauman A., Pratt M. (1998). Environmental and policy interventions to promote physical activity. Am. J. Prev. Med..

[9] Peréa F.C., Groman D., Talhami Lozano A.M., Koomas A., Sprague Martinez L.S. (2014). Urban Parks and Community Development Needs: Stories through Images Captured by Youth. Child. Youth Environ..

[10] Powers J., Tiffany J. (2006). Engaging youth in participatory research and evaluation. J. Public Health Manag. Pract..

[11] Sprague Martinez L., Richards-Schuster K., Teixeira S., Augsberger A. (2018). The Power of Prevention and Youth Voice: A Strategy for Social Work to Ensure Youths’ Healthy Development. Soc. Work.

[12] Sprague Martinez L.S., Reisner E., Campbell M., Brugge D. (2017). Participatory Democracy, Community Organizing and the Community Assessment of Freeway Exposure and Health (CAFEH) Partnership. Int. J. Environ. Res. Public Health.

[13] Cargo M., Mercer S.L. (2008). The Value and Challenges of Participatory Research: Strengthening Its Practice. Annu. Rev. Public Health.

[14] Strand K.J., Cutworth N., Stoecker R., Marullo S., Donohue P. (2003). Community-Based Research and Higher Education: Principles and Practices.

[15] Thackeray R., Hunter M. (2010). Empowering Youth: Use of Technology in Advocacy to Affect Social Change. J. Comput.-Mediat. Commun..

[16] Flanagan C., Levine P. (2010). Civic engagement and the transition to adulthood. Future Child..

[17] Strobel K., Kirshner B., O’Donoghue J., McLaughlin M. (2008). Qualities that attract urban youth to after-school settings and promote continued participation. Teach. Coll. Rec..

[18] Rose-Krasnor L. (2009). Future directions in youth involvement research. Soc. Dev..

[19] Madrigal D., Salvatore A., Casillas G., Casillas C., Vera I., Eskenazi B., Minkler M. (2014). Health in My Community: Conducting and Evaluating photovoice as a tool to promote environmental health and leadership among Latino/a youth. Prog. Community Health Partnersh..

[20] Ballard P.J., Syme S.L. (2016). Engaging youth in communities: A framework for promoting adolescent and community health. J. Epidemiol. Community Health.

[21] Ozer E.J., Douglas L. (2013). The impact of participatory research on urban teens: An experimental evaluation. Am. J. Community Psychol..

[22] Sprague Martinez L., Bowers E., Reich A.J., Ndulue U.J., Le A.A., Peréa F.C. (2016). Engaging youth of color in applied science education and public health promotion. Int. J. Sci. Educ..

[23] Sprague Martinez L.S., Reich A.J., Flores C.A., Ndulue U.J., Brugge D., Gute D.M., Peréa F.C. (2017). Critical Discourse, Applied Inquiry and Public Health Action with Urban Middle School Students: Lessons Learned Engaging Youth in Critical Service-Learning. J. Community Pract..

[24] Ozer E., Cantor J., Cruz G., Fox B., Hubbard E., Moret L. (2008). The diffusion of youth-led participatory research in urban schools: The role of the prevention support system in implementation and sustainability. Am. J. Community Psychol..

[25] Ozer E., Ritterman M., Wanis M. (2010). Participatory Action Research (PAR) in middle school: Opportunities, contraints, and key processes. Am. J. Community Psychol..

[26] Wilson N., Dasho S., Martin A., Wallerstein N., Wang C., Minkler M. (2007). Engaging young adolescents in social action through photovoice. J. Early Adolesc..

[27] Kulbok P.A., Meszaros P.S., Bond D.C., Thatcher E., Park E., Kimbrell M., Smith-Gregory T. (2015). Youths as partners in a community participatory project for substance use prevention. Fam. Community Health.

[28] Teixeira S. (2015). “It Seems Like No One Cares” Participatory Photo Mapping to Understand Youth Perspectives on Property Vacancy. J. Adolesc. Res..

[29] Teixeira S. (2016). Beyond broken windows: Youth perspectives on housing abandonment and its impact on individual and community well-being. Child Indic. Res..

[30] Ardoin N.M., Castrechini S., Hofstedt M.K. (2014). Youth–community–university partnerships and sense of place: Two case studies of youth participatory action research. Child. Geogr..

[31] Camino L.A. (2000). Youth-adult partnerships: Entering new territory in community work and research. Appl. Dev. Sci..

[32] Zeldin S., Camino L., Mook C. (2005). The adoption of innovation in youth organizations: Creating the conditions for youth–adult partnerships. J. Community Psychol..

[33] Peréa F.C., Koomas A., Sprague-Martinez L.S. Physical activity level in an urban new immigrant Latino community. Proceedings of the Robert Wood Johnson Foundation, Active Living Research Conference.

[34] Peréa F.C., Koomas A., Martinez L.S. The impact of park and playground quality and conditions and the incidence of incivilities on physical activity: Informing local efforts to promote active living. Proceedings of the Robert Wood Johnson Foundation, Active Living Research Conference.

[35] Executive Office of Workforce and Labor Development Labor Market Information. http://lmi2.detma.org/lmi/lmi_lur_a.asp.

[36] U.S. Cencus Bureau American FactFinder—Community Facts. https://factfinder.census.gov/faces/nav/jsf/pages/community_facts.xhtml.

[37] Sullivan M. (2004). City of Lawrence Open Space Plan. https://groundworklawrence.org/files/library/2004-osp-no-attachments.pdf.

[38] Groundwork Lawrence (2004). 5-Year Action Plan: Capital Projects City of Lawrence 2004 Open Space Plan [Report]. https://groundworklawrence.org/files/library/maps.pdf.

[39] McKenzie T.L., Cohen D.A., Sehgal A., Williamson S., Golinelli D. (2006). System for Observing Play and Recreation in Communities (SOPARC): Reliability and Feasibility Measures. J. Phys. Act..

[40] Lee R., Booth K., Reese-Smith J., Regan G., Howard H. (2005). The Physical Activity Resource Assessment (PARA) instrument: Evaluating features, amenities and incivilities of physical activity resources in urban neighborhoods. Int. J. Behav. Nutr. Phys. Act..

[41] Peréa F.C., Koomas A., Sprague-Martinez L.S. The Impact of Park and Playground Quality and Conditions on Physical Activity: Research to Inform Local Policies to Promote Active Living in an Urban Latino Community. Proceedings of the Robert Wood Johnson Foundation, Active Living Research Conference.

[42] Parrot R. (2004). Emphasizing “communication” in health communcation. J. Commun..

[43] Almonte K. (2010). “Appreciating Our Parks as A Community” *Caring for our Community: Getting to know our parks [*. A habitual newsletter prepared for the Lawrence community for the research project “Improving our opportunities for recreation and physical activity: Engaging youth in research and local policy advocacy”.

[44] Borrero T. (2010). “This Park Needs some TLC!” *Caring for our Community: Getting to know our parks.* A habitual newsletter prepared for the Lawrence community, for the research project “Improving our opportunities for recreation and physical activity: Engaging youth in research and local policy advocacy”.

[45] Balborda R., Flores C. (2010). Park of the Week: Howard Park. Rumbo News.

[46] Peréa F.C., Sprague Martinez L.S., Gonzalez R., Koomas A. (2011). A Report on Lawrence’s Parks [Report]. https://scholar.harvard.edu/files/flaviaperea/files/parks_report_final_.pdf.

[47] London J., Zimmerman K., Erbstein N. (2003). Youth-Led Research and Evaluation: Tools for Youth, Organizational, and Community Development. New Dir. Eval..

[48] Sprague Martinez L.S., Gute D.M., Ndulue U.J., Seller S.L., Brugge D., Peréa F.C. (2012). All public health is local. Revisiting the importance of local sanitation through the eyes of youth. Am. J. Public Health.

[49] Larson R., Walker K., Pearce N. (2005). A comparison of youth-driven and adult-driven youth programs: Balancing inputs from youth and adults. J. Community Psychol..

[50] Foster-Fishman P., Nowell B., Deacon Z., Nievar M., McCann P. (2005). Using methods that matter: The impact of reflection, dialouge, and voice. Am. J. Community Psychol..

[51] Flicker S. (2008). Who benefits from community-based participatory research? A case study for the Positive Youth Project. Health Educ. Behav..

[52] Ford T., Rasmus S., Allen J. (2012). Being useful: Acheiving indidenous youth involvement in a community-based participatory research project in Alaska. Int. J. Circumpolar Health.

[53] Rigolon A., Browning M., Jennings V. (2018). Inequities in the quality of urban park systems: An environmental justice investigation of cities in the United States. Landsc. Urban Plan..

